# Factors associated with stage of change in smoker in relation to smoking cessation based on the Korean National Health and Nutrition Examination Survey II-V

**DOI:** 10.1371/journal.pone.0176294

**Published:** 2017-05-04

**Authors:** Ah Young Leem, Chang Hoon Han, Chul Min Ahn, Sang Haak Lee, Jae Yeol Kim, Eun Mi Chun, Kwang Ha Yoo, Ji Ye Jung, Korean Smoking Cessation Study Group

**Affiliations:** 1 Division of Pulmonology, Department of Internal Medicine, Institute of Chest Disease, Severance Hospital, Yonsei University College of Medicine, Seoul, Republic of Korea; 2 Division of Pulmonology, Department of Internal Medicine, National Health Insurance Service Ilsan Hospital, Koyang, Republic of Korea; 3 Division of Pulmonology, Department of Internal Medicine, Gangnam Severance Hospital, Yonsei University College of Medicine, Seoul, Republic of Korea; 4 Division of Pulmonology, Critical Care and Sleep Medicine, Department of Internal Medicine, St. Paul's Hospital, College of Medicine, The Catholic University of Korea, Seoul, Republic of Korea; 5 Department of Internal Medicine, Chung-Ang University College of Medicine, Seoul, Republic of Korea; 6 Division of Pulmonary and Critical Care Medicine, Department of Internal Medicine, Ewha Womans University School of Medicine, Seoul, Republic of Korea; 7 Department of Internal Medicine, Konkuk University School of Medicine, Seoul, Republic of Korea; University of Oslo, NORWAY

## Abstract

Despite a decrease in incidence, smoking remains the most serious public health problem worldwide. Identification of the factors contributing to changes in willingness to quit smoking may aid the development of strategies that encourage smoking cessation. Pooled cross-sectional data from 11,924 smokers from the Korean National Health and Nutrition Examination Survey II–V were analyzed. The stages of change in smoking cessation were categorized as pre-contemplation, contemplation, and preparation. Baseline characteristics, socioeconomic factors, quality of life, psychological status, and smoking-related factors were compared between groups. The smokers were grouped as follows: 32.4% pre-contemplation, 54.4% contemplation, and 13.1% preparation. The proportion of smokers in the pre-contemplation group decreased (from 37.4% to 28.4%) from 2001 to 2012, while the proportion in the preparation group increased (from 6.4% to 18.1%). Compared with the preparation group, after adjusting for confounding factors, the pre-contemplation group was older [≥65 years-old; odds ratio (OR) = 1.40], more often single (OR = 1.38), less educated (elementary school or lower; OR = 1.93), less physically active in terms of walking (OR = 1.38) or performing strengthening exercises (OR = 1.61), smoked more heavily (≥20 cigarettes per day; OR = 4.75), and had a lower prevalence of chronic disease (OR = 0.76). Moreover, smokers who had never received education on smoking cessation were less willing to quit than those who had (OR = 0.44). In Korean smokers, the stages of change for smoking cessation were associated with age, education, marital status, chronic diseases, physical activity, and participation in smoking cessation programs.

## Introduction

Smoking is the leading cause of a number of preventable diseases such as chronic obstructive pulmonary disease, cardio-cerebrovascular disease, and malignancy. In the United States, estimates show that tobacco contributes to approximately 480,000 premature deaths and costing approximately 289 billion dollars annually owing to direct healthcare expenditure and loss of productivity [1, 2]. Despite declines in the prevalence of current smoking, the annual burden of smoking-attributable mortality in the United States has remained above 400,000 for more than a decade and currently is estimated to be about 480,000 with millions more living with smoking-related diseases [2]. Furthermore, due to the slow decline in the prevalence of current smoking, the annual burden of smoking-attributable mortality can be expected to remain at high levels for decades into the future, with 5.6 million youth currently 0 to 17 years of age projected to die prematurely from a smoking-related illness [2]. Annual smoking-attributable economic costs in the United States estimated for the years 2009–2012 were between 289–332.5 billion dollars, including 132.5–175.9 billion dollars for direct medical care of adults, 151 billion dollars for lost productivity due to premature death estimated from 2005–2009, and 5.6 billion dollars (in 2006) for lost productivity due to exposure to secondhand smoke [2].

A diversity of individual and public-based interventions aimed at smoking cessation have been developed and implemented. The Transtheoretical Model (TTM) proposed by DiClemente et al. and Prochaska et al. allows the examination of behavior associated with willingness to quit smoking with a view to developing stage-matched interventions in the United States [3–5]. The TTM can be applied to assess behavior change and provides strategies to guide the individual through the “stages of change” to action and maintenance [6]. Several health behaviors that have been applied by the TTM include smoking cessation, exercise adoption, dietary fat reduction, mammography compliance, and reduced sun exposure and alcohol consumption [6–11].

According to the TTM, smokers are classified into one of three stages of change: pre-contemplation, contemplation, or preparation [3, 12]. The stages have been redefined since they were first conceived [13]. In order to design interventions for smoking cessation, information on the distribution and characteristics of smokers in each stage is required. The stage of change is also a useful predictor of smoking cessation and intermediate indicator of movement toward smoking cessation [14, 15].

Several national, cross-sectional studies have been reported on the relationship of basic demographic or socioeconomic factors with smoking cessation [7, 10, 16–19]. Besides their inconsistent results, only few studies included various factors related to socio-economic status, health status, psychosocial attributes, and smoking-related factors together in the analysis [10]. In these studies, factors such as age, marital status, socio-economic position, health status, and smoking characteristics were analyzed [7, 10, 16–19]. In the study of prospective cohort study for Taiwanese, demographic factors and mortality were analyzed but socioeconomic factors were not included [11]. Several studies reported that physical activity may be related to tobacco use [20, 21]. However, the study which investigated the relationships between the physical activity and willingness to smoking cessation were scarce.

The aim of this study was to determine the distributions of smokers in the three stages of change in smoking cessation and to evaluate the factors contributing to changes in willingness to quit smoking in a large representative sample of the Korean population.

## Materials and methods

### Study population

We used data from the Korean National Health and Nutrition Examination Survey II–V (KHANES) to classify the three stages of change. KNHANES I–VII is an ongoing cross-sectional survey of the civilian, conducted by the Korea Centers for Disease Control [22]. The methodology of the survey is as follows in brief: A stratified multistage clustered probability design was used to select a representative sample of civilian, non-institutionalized Koreans. KNHANES I, II, III were conducted in 1998, 2001, and 2005, respectively whereas KNHANES IV (2007–2009), V (2010–2012), VI (2013–2015), and VII (2016–2018, still in progress) were conducted continuously throughout the year and contained a larger number of participants. The survey is composed of a health interview, a nutrition survey, and a health examination survey (S1, S2 and S3 Tables). Data were collected by household interviews, and standardized physical examinations and analysis of fasting blood samples were performed in mobile health examination centers [23]. After registering personal information and signing a pledge of confidentiality, anyone can download the raw data from the KNHANES website [22]. Among the subjects who participated in the survey between 2001 and 2012, we identified 11,924 smokers aged ≥20 years who reported their readiness to quit smoking. Smokers were defined by those who replied positively to the following question: “Do you smoke?”

The Korea Centers for Disease Control and Prevention obtained written and informed consent from all participants, and the Institutional Review Board of Severance Hospital approved this study protocol (4-2014-0397).

### Measures

We used data relating to two questions from the KNHANES questionnaire to classify the stages of change in smoking cessation:

Q1 Have you made a 24-hour quit attempt in the previous 12 month?
  1) Yes
  2) No
Q2 Are you planning to quit smoking within the next 1 to 6 months?
  1) Thinking of quitting smoking within the next month
  2) Thinking of quitting smoking within the next 6 months
  3) Thinking of quitting smoking at some point, but not within the next 6 months
  4) Absolutely not thinking of quitting smoking

The stages of change were defined as follows: pre-contemplation included smokers who responded to question 2 with answer 4; preparation included smokers who responded to question 2 with answer 1 and question 1 with answer 1; and contemplation included the rest.

The stages of change in smoking cessation were compared in relation to age, sex, body mass index, socioeconomic characteristics (household income, education defined by the highest level of schooling, occupation, and marital status), underlying diseases (hypertension, cardiovascular disease, and diabetes mellitus (DM)), pulmonary function test, quality of life (as assessed via the EuroQol five dimensions questionnaire (EQ-5D) questionnaire), psychological status, smoking-related factors (amount, duration, anti-smoking policy, and attendance at smoking cessation programs), alcohol use (ever-drinker; drinking more than once a month), and physical activity.

Physical activity was defined as follows. Intense physical activity included activities such as jogging, mountain climbing, cycling, swimming rapidly, playing soccer, basketball, jump rope, squash, or a tennis match of singles, at least 20 minutes, 3 times a week. Moderate physical activity included activities such as swimming slowly, playing a tennis match of doubles, volleyball, badminton, or table tennis. Muscle strengthening exercises included activities such as pushup, sit-up, dumbbell or barbell exercise, or exercising on the horizontal bar.

### Statistical analysis

Categorical variables were analyzed by using the chi-square test, and continuous variables were analyzed by using analysis of variation. Change in proportion of precontemplation, contemplation, and preparation stages over the years was tested for trend with the linear-by-linear association method. Multinomial logistic regression analysis was performed for variables that showed significance in univariate analysis to identify factors that influence the stages of change in smoking cessation. Pre-contemplation and contemplation groups were compared with the preparation group. The results are described as odds ratio (OR) with 95% confidence intervals. Statistical analysis was performed by using SAS Enterprise Guide version 9.4 (SAS Institute Inc., Cary, NC, USA). For missing data, the pairwise deletion method was used, and a *P*-value <0.05 was deemed to be statistically significant in the analysis.

## Results

### Baseline characteristics

**Fig 1:**
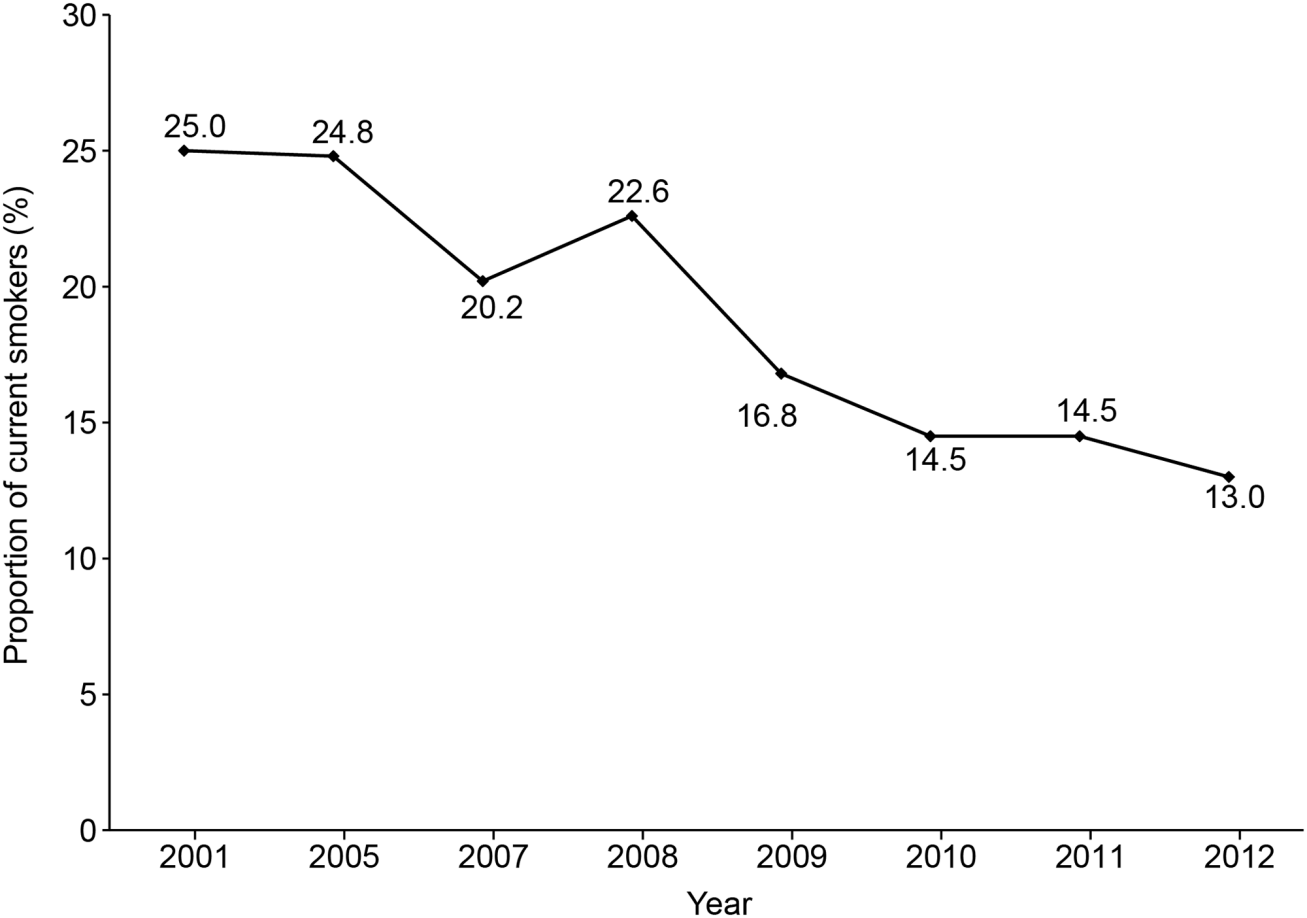
Annual percentages of current smokers in each year from 2001 to 2012.

**Fig 2:**
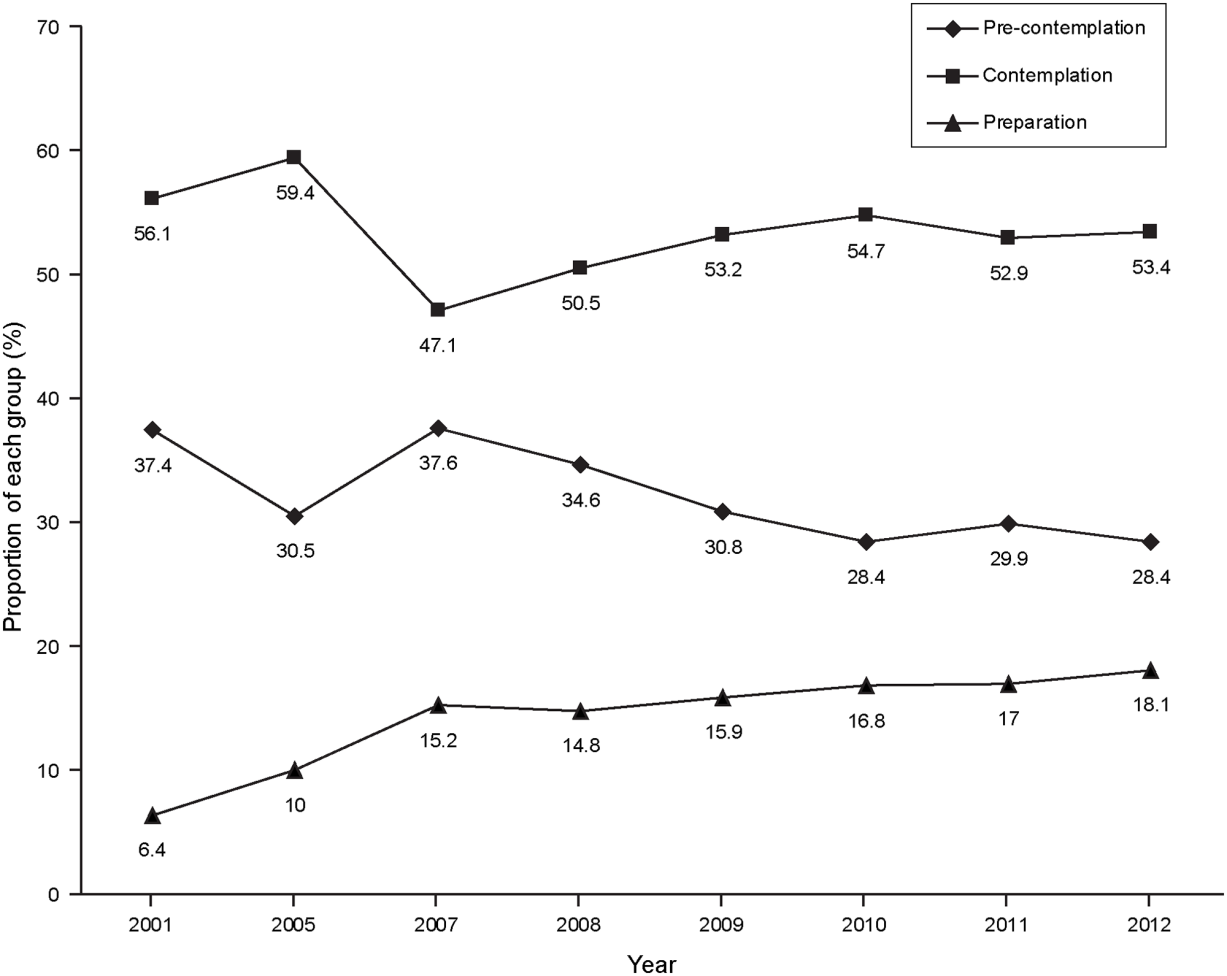
Annual percentages of the pre-contemplation, contemplation, and preparation groups in each year from 2001 to 2012.

The study included 11,924 smokers and the proportion of current smokers decreased from 2001 to 2012 (from 25.0% to 13.0%; Fig 1). Among the smokers, 32.4%, 54.4%, and 13.1% were in pre-contemplation group, contemplation group, and preparation group, respectively. While the proportion of smokers in the pre-contemplation group decreased from 2001 to 2012 (from 37.4% to 28.4%; *P*_trend_ < 0.001), the proportion in the preparation group increased (from 6.4% to 18.1%; *P*_trend_ < 0.001)(Table 1, Fig 2).

**Table 1:** Percentage of the pre-contemplation, contemplation, and preparation group for each year^a^.

Year	No.	Pre-contemplation	Contemplation	Preparation
2001	2496	934 (37.4)	1401 (56.1)	161 (6.5)
2005	2089	639 (30.5)	1241 (59.4)	209 (10.1)
2007	585	220 (37.6)	276 (47.2)	89 (15.2)
2008	1494	517 (34.6)	755 (50.6)	222 (14.8)
2009	1661	512 (30.8)	884 (53.3)	265 (15.9)
2010	1331	379 (28.5)	728 (54.7)	224 (16.8)
2011	1223	366 (29.9)	648 (53.0)	209 (17.1)
2012	1045	297 (28.4)	558 (53.4)	190 (18.2)

^a^ Data are presented as numbers (percentages)

The demographic and clinical characteristics of the study population are shown in Table 2. Compared with the contemplation and preparation groups, the pre-contemplation group was older (48.4 years vs. 42.2 years and 44.4 years; *P* <0.001), and a larger proportion was unemployed or had a blue collar job and was in the lowest household income level bracket (25.4% vs. 16.5% and 17.3%; *P* <0.001) and the lowest education level bracket (26.9% vs. 12.4% and 14.2%; *P* <0.001). The pre-contemplation group had a higher prevalence of hypertension and DM, and a lower prevalence of cardiovascular disease. Moreover, the pre-contemplation group more often had an obstructive pattern in a pulmonary function test than did the preparation group.

**Table 2:** Baseline characteristics of the pre-contemplation, contemplation, and preparation groups^a^.

Baseline characteristics	Total No. (n = 11924)	Pre-contemplation (n = 3864)	Contemplation (n = 6491)	Preparation (n = 1569)	*P*-value
Age, yrs		48.4±16.2	42.2±13.9	44.4±15.3	<0.001
<45	6584	1747 (45.2)	3955 (60.9)	882 (56.2)	<0.001
45–64	3808	1341 (34.7)	1991 (30.7)	476 (30.3)	
≥65	1532	776 (20.1)	545 (8.4)	211 (13.5)	
Sex					0.02
Male	10338	3316 (85.8)	5679 (87.4)	1343 (85.6)	
Female	1586	548 (14.2)	812 (12.6)	226 (14.4)	
BMI, kg/m^2^ ^b^		23.5±3.4	23.7±3.3	23.8±3.3	0.005
Household income					<0.001
1st quartile (lowest)	2260	951 (25.4)	1043 (16.5)	266 (17.3)	
2nd quartile	3172	1024 (27.4)	1722 (27.1)	426 (27.5)	
3rd quartile	3165	912 (24.5)	1811 (28.6)	442 (28.7)	
4th quartile (highest)	3022	850 (22.7)	1762 (27.8)	410 (26.5)	
Education					<0.001
Elementary school or lower	2051	1028 (26.9)	802 (12.4)	221 (14.2)	
Middle school	1350	511 (13.4)	680 (10.6)	159 (10.2)	
High school	4855	1439 (37.6)	2769 (42.9)	647 (41.4)	
College/university or higher	3585	849 (22.1)	2200 (34.1)	536 (34.2)	
Occupation					<0.001
White collar job^c^	4606	1228 (32.0)	2728 (42.3)	650 (41.7)	
Blue collar job^d^ or unemployed	7243	2620 (68.0)	3726 (57.7)	907 (58.3)	
Marriage					0.001
Single^e^	2840	973 (26.5)	1552 (25.6)	315 (21.6)	
Married	8361	2699 (73.5)	4518 (74.4)	1144 (78.4)	
Underlying disease^f^					
Hypertension	1403	507 (17.3)	680 (13.3)	216 (15.4)	<0.001
Dyslipidemia	554	144 (4.9)	313 (6.1)	97 (6.8)	0.071
Cerebral vascular accident	160	66 (2.2)	73 (1.4)	21 (1.4)	0.083
Cardiovascular disease	178	58 (1.9)	78 (1.5)	42 (2.9)	0.001
Pulmonary tuberculosis	497	163 (5.5)	250 (4.9)	84 (5.9)	0.469
Asthma	391	136 (4.6)	199 (3.9)	56 (3.9)	0.563
Diabetes mellitus	669	242 (8.2)	320 (6.2)	107 (7.6)	0.017
Chronic renal failure	25	8 (0.2)	15 (0.2)	2 (0.1)	0.855
Bronchiectasis	17	3 (0.1)	11 (0.3)	3 (0.3)	0.423
Depression	1048	329 (8.5)	552 (8.5)	167 (10.6)	0.02
Pulmonary function test					<0.001
FEV_1_/FVC <70%	848	326 (22.4)	386 (16.1)	136 (17.8)	
FEV_1_/FVC ≥70%	3767	1128 (77.6)	2010 (83.9)	629 (82.2)	

^a^ Categorical variables were analyzed by using the chi-square test, and continuous variables were analyzed by using analysis of variation. Data are presented as numbers (percentages) and ± values are means±standard deviation.

^b^ n = 3288 in the pre-contemplation group, n = 4432 in the contemplation group, and n = 1442 in the preparation group

^c^ White collar jobs include administrators, experts, and office workers.

^d^ Blue collar jobs include farmers, fishermen, and other manual workers.

^e^ Single includes divorced, separated, and never-married subjects.

^f^ n = 2930 in the pre-contemplation group, n = 5090 in the contemplation group, and n = 1408 in preparation group except for depression. All the underlying diseases were diagnosed by a medical provider.

BMI, body mass index; FEV_1_, forced expiratory volume in 1 second; FVC, forced vital capacity.

### Quality of life and psychological status

Table 3 shows data relating to quality of life, as assessed via the EQ-5D questionnaire, and the psychological status of the three groups. Compared with the contemplation and preparation groups, the pre-contemplation group had more problems with mobility, self-care, usual activities, and pain/discomfort. More smokers in the pre-contemplation group reported a lack of stress. However, a larger proportion of smokers in this group had experienced suicidal ideation within the past year (19.3% vs. 16.0% and 16.1%; *P* = 0.001).

**Table 3:** Quality of life and psychological status in the pre-contemplation, contemplation and preparation groups^a^.

Variables	Total No. (n = 11924)	Pre-contemplation (n = 3864)	Contemplation (n = 6491)	Preparation (n = 1569)	*P*-value
**EQ-5D**^b^					
Mobility					
No problems	8300	2470 (84.4)	4595 (90.5)	1235 (88.0)	<0.001
Problems^c^	1104	455 (15.6)	480 (9.5)	169 (12.0)	
Self-care					
No problems	9074	2784 (95.2)	4940 (97.4)	1350 (96.2)	<0.001
Problems^d^	329	140 (4.8)	135 (2.6)	54 (3.8)	
Usual activities					
No problems	8643	2607 (89.1)	4746 (93.5)	1290 (91.9)	<0.001
Problems	761	318 (10.9)	329 (6.5)	114 (8.1)	
Pain/discomfort					
No problems	7364	2221 (75.9)	4029 (79.4)	1114 (79.3)	0.001
Problems	2038	703 (24.1)	1045 (20.6)	290 (20.7)	
Anxiety/depression^e^					
No problems	8252	2543 (86.9)	4488 (88.5)	1221 (86.9)	0.068
Problems	1145	381 (13.1)	581 (11.5)	183 (13.1)	
Stress					
Yes	8144	2361 (80.6)	4544 (89.3)	1239 (88.0)	<0.001
No	1284	569 (19.4)	546 (10.7)	169 (12.0)	
Suicidal ideation within past 1 yr	1611	566 (19.3)	817 (16.0)	228 (16.1)	0.001
Suicidal attempt within past 1 yr	124	44 (1.1)	63 (0.9)	17 (1.1)	0.015
Psychiatric counseling within past 1 yr^b^	162	51 (1.7)	85 (1.6)	26 (1.8)	0.897

^a^ Categorical variables were analyzed by using the chi-square test. Data are presented as numbers (percentages).

^b^ n = 2930 in the pre-contemplation group, n = 5090 in the contemplation group, and n = 1408 in the preparation group

^c^ People who have difficulty with walking.

^d^ People who have difficulty with dressing or taking a bath.

^e^ The Anxiety and depression are based on self-report about their general mood.

### Smoking, alcohol, and physical activity

Table 4 shows data relating to smoking history, alcohol use, and physical activity. Compared with the contemplation and preparation groups, the pre-contemplation group smoked more cigarettes per day and had smoked for a longer time. Smokers in the pre-contemplation group were more often permitted to smoke in the workplace (79.8% vs. 72.7% and 68.8%; *P* = 0.013) or at home (77.8% vs. 66.4% and 60.0%; *P* <0.001) and to be never-drinkers (5.7% vs. 3.3% and 3.0%; *P* <0.001). They less often experienced smoking cessation education within past 1year (7.3% vs. 12.4% and 16.4%; *P* <0.001) and exercised or performed physical activity.

**Table 4:** Smoking, alcohol use, and physical activity between the pre-contemplation, contemplation and preparation groups^a^.

Variables	Total No. (n = 11924)	Pre-contemplation (n = 3864)	Contemplation (n = 6491)	Preparation (n = 1569)	*P*-value
**Smoking history**					
No. of cigarettes smoked per day^b^		16.9±9.2	15.2±8.0	12.1±8.1	<0.001
<10	2293	589 (15.3)	1153 (17.8)	551 (35.1)	<0.001
10–19	4473	1263 (32.7)	2637 (40.6)	573 (36.5)	
≥20	5155	2010 (52.1)	2700 (41.6)	445 (28.4)	
Duration of smoking, months^b^		288±196.1	216.1±151.9	212.1±173.1	<0.001
Attempt smoking cessation^c^					
For oneself^d^	4426	640 (76.1)	3051 (84.1)	735 (84.8)	<0.001
For others^e^	912	201 (23.9)	579 (15.9)	132 (15.2)	
Anti-smoking policy at workplace^f^					
Yes	339	69 (20.2)	227 (27.3)	43 (31.2)	0.013
No	971	273 (79.8)	603 (72.7)	95 (68.8)	
Anti-smoking policy at home^g^					
Yes	631	140 (22.2)	411 (33.6)	80 (40.0)	<0.001
No	1423	490 (77.8)	813 (66.4)	120 (60.0)	
Existence of a regular smoker at home^f^	3018	1063 (36.2)	1648 (32.3)	307 (21.8)	<0.001
Smoking cessation education experience within past 1 yr^g^	7339	167 (7.3)	478 (12.4)	197 (16.4)	<0.001
**Alcohol use**^f^					
Ever-drinker^h^	9049	2763 (94.3)	4921 (96.6)	1365 (96.9)	<0.001
Never-drinker	379	167 (5.7)	169 (3.3)	43 (3.0)	
**Physical activity**^f^					
Number of days of intense physical activity^i^ (per week)					
None	5882	2009 (68.5)	3058 (60.0)	815 (57.8)	<0.001
One day or more	3543	921 (31.4)	2031 (39.9)	592 (42.1)	
Number of days of moderate physical activity^j^ (per week)					
None	5268	1832 (62.4)	2700 (53.0)	738 (52.4)	<0.001
One day or more	4154	1098 (37.4)	2389 (46.9)	669 (47.6)	
Number of days of walking (per week)					
None	1247	522 (17.8)	565 (11.1)	160 (11.3)	<0.001
One day or more	8179	2408 (82.2)	4524 (88.9)	1247 (88.6)	
Number of days of muscle strengthening exercises^k^ (per week)					
None	6604	2226 (75.9)	3519 (69.1)	859 (61.0)	<0.001
One day or more	2822	704 (24.0)	1570 (30.9)	548 (38.9)	

^a^ Categorical variables were analyzed by using the chi-square test, and continuous variables were analyzed by using analysis of variation. Data are presented as numbers (percentages) and ± values are means±standard deviation.

^b^ n = 3862 in the pre-contemplation group, n = 6491 in the contemplation group, and n = 1569 in the preparation group

^c^ n = 841 in the pre-contemplation group, n = 3630 in the contemplation group, and n = 867 in the preparation group

^d^ Self-awareness or concern for their own health.

^e^ Concern or request from other people.

^f^ n = 2930 in the pre-contemplation group, n = 5089 in the contemplation group, and n = 1407 in the preparation group

^g^ n = 2291 in the pre-contemplation group, n = 3849 in the contemplation group, and n = 1199 in the preparation group

^h^ Drinking more than once a month

^i^ Intense physical activity includes activities such as jogging, mountain climbing, cycling, swimming rapidly, playing soccer, basketball, jump rope, squash, or a tennis match of singles, at least 20 minutes, 3 times a week.

^j^ Moderate physical activity includes activities such as swimming slowly, playing a tennis match of doubles, volleyball, badminton, or table tennis

^k^ Muscle strengthening exercises include activities such as pushup, sit-up, dumbbell or barbell exercise, or exercising on the horizontal bar

### Multivariate analysis

Multivariate analysis was performed for variables that showed significance in univariate analysis (Table 5). Relative to the preparation group, the pre-contemplation group was older (≥65 years of age; OR = 1.40), less educated (elementary school or lower; OR = 1.93), more often single (OR = 1.38) and anxious or depressed (OR = 0.75), smoked more heavily (≥20 cigarettes per day; OR = 4.75), and had a lower level of physical activity in terms of walking (OR = 1.38) or performing strengthening exercises (OR = 1.61) and a lower prevalence of chronic diseases (hypertension, DM, or cardiovascular disease)(OR = 0.76). Moreover, smokers without smoking cessation education experience within past 1 year were less willing to quit smoking (OR = 0.44). Compared with the preparation group, the contemplation group contained more women (OR = 0.79), heavy smokers (OR = 2.98), and single individuals (OR = 1.26), fewer individuals with previous chronic disease (OR = 0.82), and fewer physically active individuals (OR = 1.42), and fewer individuals with smoking cessation education experience within past 1 year (OR = 0.70).

**Table 5:** Multivariate analysis for factors associated with stage of change in smoking cessation^a^.

Variables	Pre-contemplation (n = 2025)	Contemplation (n = 3338)
**Demographic factors**		
Age, yr		
45–64 (vs. <45)	1.10 (0.90–1.34)	1.02 (0.85–1.23)
≥65 (vs. <45)	1.40 (1.03–1.89)	0.77 (0.58–1.03)
Sex (male vs. female)	0.83 (0.64–1.07)	0.79 (0.63–0.99)
Underlying diseases		
Chronic disease^e^ (vs. none)	0.76 (0.63–0.93)	0.82 (0.68–0.98)
Depression (vs. none)	0.81 (0.61–1.08)	1.03 (0.79–1.35)
**Social factors**		
Household income		
1st quartile (vs. 4th quartile)	1.03 (0.78–1.36)	0.94 (0.72–1.22)
2nd quartile (vs. 4th quartile)	0.93 (0.75–1.17)	0.84 (0.69–1.03)
3rd quartile (vs. 4th quartile)	0.86 (0.69–1.07)	0.91 (0.75–1.10)
Education		
Elementary school or lower (vs. college/university or higher)	1.93 (1.42–2.63)	0.93 (0.70–1.25)
Middle school (vs. college/university or higher)	1.44 (1.07–1.94)	0.87 (0.66–1.15)
High school (vs. college/university or higher)	1.24 (1.01–1.53)	0.94 (0.79–1.13)
Occupation		
White collar job^b^ (vs. blue collar job^c^ or unemployed)	0.96 (0.79–1.16)	0.94 (0.79–1.11)
Marital status		
Single^d^ (vs. married)	1.38 (1.12–1.70)	1.26 (1.04–1.53)
**Smoking and alcohol related factors**		
No. of cigarettes smoked per day		
10–19 (vs. <10)	2.59 (2.10–3.20)	2.21 (1.84–2.65)
≥20 (vs. <10)	4.75 (3.82–5.93)	2.98 (2.46–3.62)
Never-drinker (vs. ever drinker^f^)	1.38 (0.91–2.10)	1.32 (0.88–1.99)
**Life style factors**		
Suicidal ideation within past 1 yr (vs. none)	1.20 (0.95–1.52)	1.08 (0.87–1.35)
Smoking cessation education experience within past 1yr (vs.none)	0.44 (0.34–0.56)	0.70 (0.57–0.86)
Number of days of walking exercise (per week)		
None (vs. 1 day or more)	1.38 (1.11–1.72)	1.01 (0.82–1.25)
Number of days of strengthening exercise^g^ (per week)		
None (vs. 1 day or more)	1.61 (1.35–1.01)	1.42 (1.22–1.66)
EQ-5D		
Mobility, problems^h^ (vs. no problems)	0.91 (0.67–1.23)	0.92 (0.69–1.24)
Self-care, problems^i^ (vs. no problems)	0.85 (0.54–1.32)	0.93 (0.60–1.43)
Usual activities, problems (vs. no problems)	1.04 (0.71–1.52)	0.96 (0.67–1.39)
Pain/discomfort, problems (vs. no problems)	1.06 (0.84–1.33)	1.05 (0.84–1.30)
Anxiety/depression, problems (vs. no problems)	0.75 (0.56–0.99)	0.81 (0.62–1.05)

^a^ Multinomial logistic regression analysis was performed to identify factors that influence the stages of change in smoking cessation. Pre-contemplation and contemplation groups were compared with the preparation group. Data are presented as odds ratios (95% confidence intervals) compared with the preparation group (n = 1063).

^b^ White collar job includes administrators, experts, and office workers.

^c^ Blue collar job includes manual workers such as farmers, fishermen, and others.

^d^ Single includes divorced, separated, and never married subjects.

^e^ Chronic disease includes hypertension, diabetes mellitus, or cardiovascular accident.

^f^ Drinking more than once a month.

^g^ Muscle strengthening exercises include activities such as pushup, sit-up, dumbbell or barbell exercise, or exercising on the horizontal bar.

^h^ People who have difficulty with walking.

^i^ People who have difficulty with dressing or taking a bath.

EQ-5D, EuroQol five dimensions questionnaire

## Discussion

This study demonstrated significant associations between the stages of change in smoking cessation in the Korean population and the following: age, education level, marital status, cardiovascular disease, amount of smoking, physical activity, and education regarding smoking cessation. Our study population comprised 32.4% in the pre-contemplation group, 54.4% in the contemplation group, and 13.1% in the preparation group. While the proportion of smokers in the pre-contemplation group decreased from 2001 to 2012, the proportion in the preparation group increased.

The stages of change have been redefined since their conception was first introduced in 1984 [3, 12]. Initially, there were five stages (pre-contemplation, contemplation, action, maintenance, and relapse) [5, 9]. Preparation was added by DiClemente et al. in 1991 [3], and smokers were then divided into three groups of pre-contemplation, contemplation, or preparation groups [3, 7, 11, 12, 17]. In those studies, relapse was considered as an event that terminates the other phases prompting a cyclical movement back through the initial stages, not a different stage [3, 7, 10–12, 17]. We used data from the KNHANES to classify the three stages of change, in accordance with previous reports as follows: the pre-contemplation stage includes smokers who do not seriously consider quitting within the next 6 months, and the preparation stage includes smokers who “intend to quit” or “seriously think about quitting” in the next 30 days and have made a 24-hour quit attempt in the previous 12 months. The rest were classified as the contemplation stage [3, 7, 11, 12, 17].

### Distribution of smokers according to stages of change for smoking cessation

The distribution of smokers according to stages of change for smoking cessation has been investigated in a number of countries at different times with different definition of stage of change. Mbulo et al. used Global Adult Tobacco Survey data to categorize smokers into different stages of change [7]. In this paper, most smokers across all countries were in the pre-contemplation stage, followed in order by the contemplation stage and the preparation stage [7]. Wewers et al. reported that 59.1%, 33.2%, and 7.7% of American respondents were in the pre-contemplation, contemplation, and preparation stages, respectively, in 1992–1993 [18]. Subsequent surveys in the United States showed similar distributions in 1995–1996 and 1998–1999 [18]. In thet study by Cambell et al. between 2003–2004, 39.6%, 43.4%, and 13.9% of 594 Australians were assigned to the pre-contemplation, contemplation, and preparation groups, respectively; these values are similar to those in our study of the Korean population [16]. Daoud et al. reported that 61.8%, 23.8%, 14.4% were in the pre-contemplation, contemplation stage, and preparation stage, respectively, among Arab men between 2012–2013 [10]. The preparation group showed the least proportion of less than 15% in all studies. However, different distributions between studies might be attributed to differences in definition, race, culture, study duration, and local anti-smoking policies [7, 16, 18]. Wewers et al used similar definitions that we did while Cambell et al. and Daoud et al. used broader definition for preparation group. Moreover, the age was limited to less than 65 years old in the study of Daoud et al. [7, 16, 18].

To promote public health, the Korean government implemented various anti-smoking services and policies after the National Health Promotion Act was announced in 1995 [24, 25]. These include the designation of public places as non-smoking areas and the curtailment of cigarette advertisements. A nationwide smoking cessation program was introduced in 253 health centers in 2005, and free nicotine replacement therapy and individual counseling were provided. In addition, small-scaled nationwide quitline smoking cessation services were initiated in 2006, and the price of cigarettes was increased nearly twofold in 2015. These efforts might be related with augmenting the smoker’s willingness to quit smoking in Korea.

### Factors associated with the stages of change in smoking cessation

Most of the studies that examined the demographic and socioeconomic factors associated with the stages of change in smoking cessation reported socioeconomic status as significant [17–19]. According to age-stratified analyses, smoking characteristics such as number of cigarettes smoked, duration of smoking, and age at smoking onset were also related to the stages of change [17]. Our study provides further evidence that as levels of education and income increase, the proportion of smokers in the pre-contemplation group decreases, and the proportions in the contemplation and preparation groups increase. Moreover, those who had smoked for a longer time and who smoked more cigarettes per day were more likely to be in the pre-contemplation group. Among the smokers who attempted to quit, more attempted for themselves than for others, although more smokers in the pre-contemplation group attempted to quit for others. In this context, being single was another important factor influencing the stage of change in smokers.

Psychiatric disorders, such as major depressive disorders and anxiety disorders, are known to increase the risk of smoking and decrease the likelihood of quitting [26–28].: Stanton et al. reported that conditions such as anxiety, depression, and substance abuse were related to higher tobacco use, although proportion of cigarette smoking declined significantly over time among adults with no chronic condition [29]. Anxiety, stressful life events, and social support were psychological factors relevant to the stages of changes, as reported by Daoud et al. [10]. In our study, although the proportion of people diagnosed with depression was lower in the pre-contemplation group, levels of suicidal ideation and attempted suicide were higher. According to the EQ-5D questionnaire, smokers in the pre-contemplation group were less anxious and depressed; however, this questionnaire might be too simplistic to accurately evaluate the severity and type of anxiety or depression. Moreover, those with a low level of physical activity, which is associated with an increased likelihood of depression, were less willing to quit [30]. In the study of Loprinzi et al. maintenance of regular physical activity among young daily smokers may help to facilitate smoking cessation [31]. However, the relationship between physical activity and willingness to quit smoking has not been evaluated clearly.

Progression through the stages of change in smoking cessation requires changes in knowledge, attitude, and beliefs in regard to smoking, particularly for those who are unwilling to attempt quitting [16]. Our study suggests that additional effort is required for the elderly, those with a lower level of education or exercise, those without chronic disease or smoking cessation education experience, those who are single or anxious/depressed, or those who smoked more heavily in pre-contemplation stage. In comtemplation stage, additional effort is required for women, heavy smokers, singles, those without chronic disease, those without smoking cessation education experience, or those with low levels of exercise. Both individual and public health approaches are needed to increase quitting rates. On an individual level, clinicians can encourage smokers to quit by presenting the potential benefits of quitting, the harmful effects of smoking, and the coping strategies for barriers to quitting [32, 33]. Education in regard to smoking cessation was strongly associated with change in our study. Similarly, the stages of change were related to knowledge of the hazards of smoking and a positive attitude toward smoking prevention in a study of current smokers by Daoud et al. [10] and to smoking-related thoughts such as the pros and cons of smoking, the temptation to smoke, and the ability to resist smoking in a study of Taiwanese male smokers by Luh et al. [11]. However, all smokers coming for smoking cessation programs are not ready for action and they should be grouped according to which stage of change they are in. The interventions vary according to smoker’s individual stages and using the ten processes of change previously presented could help to identify strategies specific to each stage leading to individualized approach [5, 34]. Public health approaches include increases in cigarette taxes, ban smoking in public places, smoking cessation campaigns with prohibition of tobacco advertising, and the placement of warnings or pictures on cigarettes products [35]. According to our analysis, anti-smoking policies in the workplace and at home have led to smokers to become more willing to quit.

Unlike previous studies, we considered marital status, anti-smoking policies in the workplace and smoke-free rules in homes, anti-smoking education, psychological status, physical activity, and quality of life in our analyses. The information obtained will facilitate the development of interventions that encourage progression through the stages of change in smoking cessation and will allow strategies to be tailored to the characteristics of the smoker.

### Limitations

This study has several limitations. First, it was a cross-sectional and observational study, and thus it is difficult to clarify the cause-and-effect relationships between the associated factors and the stages of change in smoking cessation. However, the large study population might compensate for the methodological bias. Moreover, data for multiple years were integrated in one data set, but adjustment of years of survey did not show differences. Longitudinal cohort study is warranted to overcome this limitation. Second, data on smokers who had since quit were not available; this information is important in identifying the type of smokers that will actually quit. However, the aim of this study was to evaluate the factors that contribute to changes in the willingness of smokers to stop smoking, and this analysis was accomplished by comparing pre-contemplation, contemplation, and preparation groups. Third, the questions related with stage of change in smoking cessation in this study are not exactly in accord with previous traditional criteria [3, 36]. KNHANES is designed to assess the health and nutritional status of Koreans, so other previous definitions which can be applied with questions in KNHANES were searched and used in this study [3, 11, 12, 17]. Owing to differences in the definition of the stages of change, it may be difficult to directly compare the results of all relevant studies. Fourth, the level of nicotine dependence is an important factor in smoking cessation, and there are commonly used measures of nicotine dependence such as Fagerström Tolerance Questionnaire, Fagerström Test for Nicotine Dependence and Heaviness of Smoking Index. [37–39] Although the number of cigarettes per day is an important indicator for nicotine dependence, other variables related to nicotine dependence scales were not available in the KNHANES. Lastly, we used the pairwise deletion method to deal with missing data, so each computed statistic may be based on a different subset of cases. However, the missing number in the final model was 1135 (9.5% of total population), and the distributions of the important variables were not significantly different between total population and subpopulation in the final model.

## Conclusions

In conclusion, relative to the preparation group, the pre-contemplation group was older, less educated, more often single and anxious or depressed, smoked more heavily, and had a lower level of physical activity in terms of walking or performing strengthening exercises and a lower prevalence of chronic diseases (hypertension, DM, or cardiovascular disease). Moreover, smokers without smoking cessation education experience within past 1 year were less willing to quit smoking. Compared with the preparation group, the contemplation group contained more women, heavy smokers, and single individuals. Moreover, fewer smokers in the contemplation group had chronic diseases, were physically active, and experienced smoking cessation education within past 1 year. Future interventions to encourage smokers to move from the pre-contemplation to contemplation and preparation stages of smoking cessation should take these factors into consideration.

## Supporting information

S1 Table Survey contents of KNHANES—Health examinations. (DOC)

S2 Table Survey contents of KNHANES—Health interview. (DOC)

S3 Table Survey contents of KNHANES—Nutrition survey. (DOC)

## Abbreviations

BMI: body mass index
EQ-5D: EuroQol five dimensions questionnaire
FEV1: forced expiratory volume in 1 second
FVC: forced vital capacity
KNHANES: Korean National Health and Nutrition Examination Survey
OR: odds ratio
