# Supplementary material for: Factors associated with stage of change in smoker in relation to smoking cessation based on the Korean National Health and Nutrition Examination Survey II-V
Source: PLoS One. 2017 May 4;12(5):e0176294. doi: 10.1371/journal.pone.0176294 (PMC5417445; doi:10.1371/journal.pone.0176294)
Supplement: S1 Table — (DOC) [file pone.0176294.s001.doc]

**Supplementary Table 1.** **Survey contents of KNHANES – Health Examinations**

| **Examination** | | **Examination items** |
| --- | --- | --- |
| **Anthropometry** | | Height |
|  |  | Weight |
|  |  | Waist circumference |
| **Blood pressure measurements** | | Systolic blood pressure, diastolic blood pressure |
|  |  | Pulse |
| **Muscular strength test** | | Muscular strength measurement |
| **Blood test** | **Dyslipidemia** | Total cholesterol |
|  |  | Triglyceride (TG) |
|  |  | High density lipoprotein cholesterol (HDL) |
|  |  | Low density lipoprotein cholesterol (LDL) |
|  | **Renal function** | Blood urea nitrogen (BUN) |
|  |  | Creatinine |
|  | **Diabetes** | Fasting blood sugar (FBS) |
|  |  | Glycated hemoglobin A1c (HbA1c) |
|  | **Liver diseases** | Hepatitis B surface antigen (HBsAg) |
|  |  | Hepatitis C antigen |
|  |  | Hepatitis C RNA |
|  |  | GOT |
|  |  | GPT |
|  | **Anemia** | Hemoglobin |
|  |  | Hematocrit |
|  | **General test** | Red blood cell count |
|  |  | White blood cell count |
|  |  | Vitamin D |
|  | **Thyroid diseases** | Thyroid-stimulating hormone (TSH) |
|  |  | Free T4 |
|  |  | Anti-thyroid peroxidase antibody (TPO Ab) |
| **Urine test** | **Thyroid diseases** | Iodine |
|  | **Renal function** | Microalbumine |
|  | **General test** | Urine protein |
|  |  | Urine glucose |
|  |  | Urine occult blood |
|  |  | Urobilinogen |
|  |  | Ketone |
|  |  | Bilirubin |
|  |  | Specific gravity |
|  |  | Urine PH |
|  |  | Urine nitrate |
|  |  | Urine creatinine |
|  |  | Urine cotinine |
|  |  | Urine natrium |
| **Oral health examination** | | Dentition status(dental caries, enamel fluorosis, denture status) |
|  |  | Periodontal status(community periodontal index) |
| **Spirometry** | | Chronic obstructive pulmonary disease (COPD) |
| **Eye examination** | | Visual acuity and refraction test, Dyschromatopsia |
| **Ear-nose-and-throat examination** | | Chronic sinusitis, exposure to noise, etc. |
