# Supplementary material for: Factors associated with stage of change in smoker in relation to smoking cessation based on the Korean National Health and Nutrition Examination Survey II-V
Source: PLoS One. 2017 May 4;12(5):e0176294. doi: 10.1371/journal.pone.0176294 (PMC5417445; doi:10.1371/journal.pone.0176294)
Supplement: S2 Table — (DOC) [file pone.0176294.s002.doc]

**Supplementary Table 2.** **Survey contents of KNHANES – Health Interview**

| **Subjects** | **Contents** |
| --- | --- |
| **Household survey** | sex, age, marital status, number of householders, household type, household income, health insurance, nationality, dementia diagnosis |
| **Adult** | medical conditions, health care utilization, medical examination and vaccination, activity limitation, quality of life, Injury, physical activity, mental health, education and economic activities, women health |
|  | smoking, alcohol use, obesity and weight control, safety, mental health (sleeping time, perceived stress), oral health |
| **Adolescent** | medical conditions, health care utilization, vaccination, activity limitation, Injury, physical activity, education and economic activities, women health |
|  | smoking, alcohol use, obesity and weight control, safety, mental health, oral health |
| **Children** | medical conditions, health care utilization, vaccination, activity limitation, Injury, women health |
|  |  |
|  | Obesity and weight control, safety, oral health, education |
|  |  |
