# Supplementary material for: Factors associated with stage of change in smoker in relation to smoking cessation based on the Korean National Health and Nutrition Examination Survey II-V
Source: PLoS One. 2017 May 4;12(5):e0176294. doi: 10.1371/journal.pone.0176294 (PMC5417445; doi:10.1371/journal.pone.0176294)
Supplement: S3 Table — (DOC) [file pone.0176294.s003.doc]

**Supplementary Table 3.** **Survey contents of KNHANES – Nutrition Survey**

| **Survey** | **Contents** |
| --- | --- |
| **Dietary behavior** | Skipping frequency of breakfast, lunch, evening meal |
|  | Frequency of eating out |
|  | Accompanying meal |
|  | Experience in dietary supplement use |
|  | Experience in food assistance recipients |
|  | Experience in nutrition education and consultation |
|  | Nutrition labelling : use, nutrient of interest, effect on food choice |
|  | Breast-feeding : yes or no, period |
|  | Formula-feeding : yes or no, period |
|  | Complementary feeding, milk : the time of beginning |
| **Food intake** | Name and amount of food intake in a day, name and amount of ingredients of home cooked dishes |
| **Food frequency** | Food frequency and intake amount for 112 food items |
| **Food security** | Household food security status |
